# The Effect of Lead Exposure on Autism Development

**DOI:** 10.3390/ijms22041637

**Published:** 2021-02-06

**Authors:** Aanya Goel, Michael Aschner

**Affiliations:** Department of Molecular Pharmacology, Albert Einstein College of Medicine Bronx, New York, NY 10461, USA; aagoelsr22@herricksk12.org

**Keywords:** lead, autism, neurotoxicity

## Abstract

Autism Spectrum Disorder (ASD) remains one of the most detrimental neurodevelopmental conditions in society today. Common symptoms include diminished social and communication ability. Investigations on autism etiology remain largely ambiguous. Previous studies have highlighted exposure to lead (Pb) may play a role in ASD. In addition, lead has been shown to be one of the most prevalent metal exposures associated with neurological deficits. A semi-systematic review was conducted using public databases in order to evaluate the extent of lead’s role in the etiology of autism. This review examines the relationship between autistic comorbid symptoms—such as deterioration in intelligence scores, memory, language ability, and social interaction—and lead exposure. Specifically, the mechanisms of action of lead exposure, including changes within the cholinergic, dopaminergic, glutamatergic, gamma aminobutyric acid (GABA)ergic systems, are discussed. The goal of this review is to help illustrate the connections between lead’s mechanistic interference and the possible furthering of the comorbidities of ASD. Considerations of the current data and trends suggest a potential strong role for lead in ASD.

## 1. Introduction

Autism Spectrum Disorder (ASD) is a neurodevelopmental condition characterized by deterioration in communication and social interaction. Cognitive and occupational impairment may occur as well. Autism has become one of the most common disorders among children. In 2016, 1 in 54 children in the United States had been diagnosed with ASD according to the Center of Disease Control (CDC) [1]. On average, 1 in 160 children worldwide develop ASD. Due to increased access to healthcare surveillance, the prevalence of ASD has increased in recent years. According to the Autism and Developmental Disabilities Monitoring Network (ADDM), autism prevalence increased by 78% between 2002 and 2008 in the U.S. [2]. Common symptoms include repetitive behaviors, difficulty in memory and focus, lowered cognitive ability, deficits in language, inability to distinguish feelings, and limited eye contact [3,4].

ASD is associated with intense social and financial burdens. A recent study identified an average 14% loss of income for parents of autistic children within the U.S. [5]. Reduction in work hours due to child’s status and additional medical care services are also common among families with autistic children. Extra support services and special education assistance may be necessary, further contributing financial tension [5]. Individuals with autism are more likely to be subjected to discrimination involving insufficient access to health care and educational opportunities.

ASD continues to be a health concern, as the origin of autism remains uncertain [6]. Genetics, environment, and heightened vulnerability have all been considered to play a role in the initiation of autism [1]. Previous studies have shown exposure to heavy metals such as lead (Pb) may contribute to the comorbidities that are associated with ASD [6]. Lead, a malleable metal, is found within the carbon group [7]. Common sources of lead toxicity include exposure to polluted water and soil, fertilizers, lead pipes, industrial facilities, and contaminated paint and gasoline in older housing. Houses constructed earlier than 1978 may still have traces of lead paint [6]. Children may be at higher risk for lead exposure due to prenatal exposure as well as proximity to toys containing lead [6]. An ordinary blood test obtained from a vein can be used to measure the amount of lead in the body. Children should be regularly tested for lead poisoning between the ages of one and two at the recommendation of the American Academy of Pediatricians. Normally, lead levels are measured in terms of micrograms per deciliter (μg/dL) [8].

The adverse health consequences of lead should be investigated in the context of its long half-life and moderate turnover within the body [6]. Lead primarily enters the body through ingestion (digestive system) and inhalation (breathing/respiratory system). Miniscule amounts may also enter via the skin [9]. Lead infiltrates the bloodstream and typically is excreted in urine, sweat, and saliva [9]. Excess lead binds to red blood cells and is dispersed to various soft tissues including the brain and kidneys. Lead also deposits in the bone, from which it may reenter the bloodstream upon increased osteoblastic activity, such as during pregnancy, aging, and menopause [7]. Furthermore, the typical half-life of lead in bone is 20–30 years. Prior to 2012, 10 μg/dL and above of blood lead levels was the normal level of concern. The CDC has updated its requirements about the lead level of concern. Currently, there are no safe blood lead levels, and minute traces of blood lead are considered harmful, increasing the concern regarding lead exposure [10].

**Figure 1:**
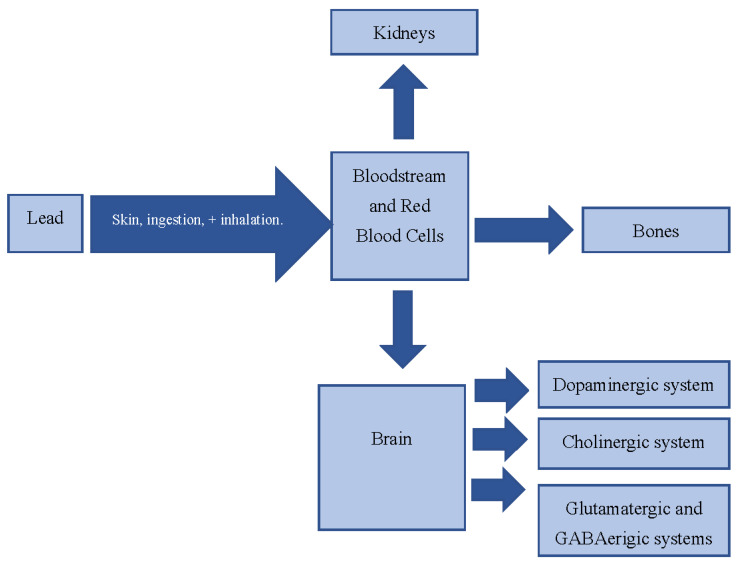
Representation of the routes of lead in the body.

Previous studies have investigated the possible association between lead exposure during childhood development and cognitive decline and behavioral issues [11,12]. Lowered test scores, deficits in attention and memory, as well as difficulties with language are common among children chronically exposed to lead [12]. Previous studies have examined how lead may induce alterations in several neurological pathways that may be responsible for these comorbidities. These changes include adjustments in the cholinergic, glutamatergic, and dopaminergic systems, along with modifications of several enzymes including protein kinase C and choline acetyltransferase [11,12]. The remainder of this review concentrates on the connection between lead exposure and the possible comorbidities of autism. Figure 1 includes a representation of lead’s means of entrance within the body.

## 2. Methods

The focus of this study was to determine the prevalence of autism in relation to lead exposure and to explore the mechanisms contributing to autistic development. Due to the nature of this topic, this study was conducted as a semi-systematic review in order to enable a more narrative approach. The development of autism and its prevalence in terms of lead exposure have been studied by several groups of researchers utilizing various methods; therefore, a narrative approach is used to describe the most recent scientific findings and their progression over time. Overall, a thematic analysis was carried out in which trends and patterns were analyzed to identify the most significant connections between lead exposure and autism. This review was designed to be realized from home, while complying with social distancing. Public databases including Science Direct and PubMed were the primary sources for accessing journals. Primarily, online journals and published texts were utilized in this study. Additionally, all sources were documented using Zotero, an online reference management platform. Prior to distinguishing specific areas of focus within the review, background research was conducted on autism and its primary symptoms to make adequate connections to the effects of lead exposure. Consistent symptoms acknowledged in these preliminary articles were decrements in intelligence scores, lack in communication ability, social withdrawal, as well as memory loss [1,2].

Subsequent to determining the most prevalent symptoms, preliminary research was conducted on the general impact of lead exposure on neurological processes as well. Within these preliminary articles, concurrence of the negative impact of lead exposure in childhood and the development of academic as well as social difficulties was found [12,13,14]. Next, a literature selection criterion was created for the further research of journal articles. Specifically, articles were chosen based on their relevance to lead exposure and decrements in academic or social ability. The majority of these articles highlighted diminution in memory, intelligence scores, social interaction, as well as language proficiency. Therefore, articles were specially chosen in relevance to lead exposure and the prevalence of the four previously mentioned topics. Subsequent to evaluating the prevalence of autistic symptoms subsequent to lead exposure, research on the mechanistic impact of lead was evaluated. Within these articles, significant transformations within the cholinergic, glutamatergic, and dopaminergic systems were distinguished [7,11]. Thus, studies including information on the effect of lead on aspects of these systems were also selected. After researching the mechanistic portion, a general organization of this review was written. Sections on the discussion of the prevalence of ASD in terms of lead exposure and the mechanisms of action were to be included. During the writing of this review, significant data from the articles researched were included based upon their relevance to the focus areas of this review (including decrements in intelligence scores, memory, social skills, language ability). Data were also included to discuss the recent changes observed within the dopaminergic, cholinergic, and glutamatergic systems subsequent to lead exposure.

## 3. The Association between Lead Exposure and the Comorbidities of Autism

### 3.1. Intelligence Scores

Excessive lead exposure may have various consequences on the development of children, including deficits in cognitive ability, memory, attention, as well as language [7,13]. Previous studies have found that increased blood lead levels have been inversely associated with cognitive and intelligence scores. One study measured the blood lead concentrations of 172 children between the ages of 6 and 60 months. Each child took a Stanford–Binet Intelligence Scale Intelligence Quotient (IQ) between the ages of 3 and 5 years. An increase of 10 μg per deciliter in the lifetime average blood lead concentration was correlated with a 4.6-point decline in IQ scores [14]. Another study specifically evaluated the effect of postnatal exposure on intelligence scores of children. A total of 1502 pregnant women in Yugoslavia were categorized by the town they lived in; one town was based around a lead smelter, while the other town involved little to no exposure to lead [15]. Blood lead levels of each child were measured every 6 months following delivery. Additionally, cognitive function assessments were carried out at least once between the ages of 3 and 7 years. An average 2.82 IQ-point loss corresponded to a 50% elevation in blood lead levels [15]. Predominantly, studies have found an average reduction of 1–3 IQ points subsequent to an increase in 10–15 μg per deciliter in blood lead concentration [7,13,14,15]. These findings suggest an inverse relationship between increased doses of lead exposure and intelligence scores. However, it should be noted that these effects are comorbidities, and their presence does not necessarily implicate that the individual has autism.

### 3.2. Memory

Previous research has shown decreased memory performance in most lead-exposed adults. [16,17,18,19,20,21]. One study determined the neurological ramifications of inserting lead acetate into the drinking water of rats. In this work, 54.7 mg/L lead acetate was instilled into the drinking water of 16 rats. The spatial memory of rats was tested using the Morris Water Maze task, in which animals must find a hidden platform within an opaque pool. During the Morris Water Maze task, lead-exposed rats showed a greater latency to reach the platform. Lower spatial memory scores suggest lead may play a role in altering spatial memory and learning. Previous studies have also investigated how lead impacts human memory. In 1982, a cohort study of lead-exposed workers found that adults (aged 55 years and older) with current mean blood lead levels of 40 μg/dL performed significantly worse than their non-exposed counterparts with current mean blood levels of 7.2 μg/dL on learning and memory assessments including the Pittsburgh Occupational Exposures Test battery. The beta coefficients of the memory scores were evaluated, and it was found that in lead-exposed individuals beta coefficients were five times higher compared to non-exposed individuals (statistically, the higher the absolute value of the beta coefficient, the greater the effect). Additionally, lead-exposed adults, aged 55 and older, experienced a 17% greater decline in cognitive score compared to controls [22]. Despite a higher risk in older individuals, memory loss following lead exposure was also prevalent in younger individuals.

Another study evaluated memory performance and retrieval of information ability using the Rey Auditory Verbal Learning Test (RAVLT). Two hundred fifty-six lead smelter workers with a current mean blood level of 28.8 μg/dL and mean age of 41 years repeated words after 15-word lists were presented to them. Lead-exposed workers had significantly increased difficulty repeating the words correctly compared to non-exposed individuals [16], further supporting that lead exposure may alter memory and learning function. This also suggests that lead exposure may play a role in inducing memory loss, which is a comorbidity of autism.

### 3.3. Language

In addition to decrements in intelligence scores, social interaction, and memory, deficits in reading and language capabilities have been common among lead-exposed individuals [16,17,18,19,20,21]. Another study conducted using the National Health and Nutrition Examination Survey (NHANES) estimated 1,025,695 people in the U.S. were born with listening, speaking, reading, writing, or reasoning disabilities from 1989 to 1998 due to escalating blood lead levels [23]. Most children experience difficulty in developing language skills following lead exposure [17]. One study evaluated the relationship between infant lead exposure and primary development using the Bayley Scales of Infant Development assessments. High-lead-exposed (with a mean blood lead level >10 μg/dL) infants scored an average of 7.3 points lower than low-lead- exposed children (with a mean blood lead level <3 μg/dL) on mental development index scores. In language, specifically, children exposed to greater amounts of lead scored an average 1 point lower than children exposed to smaller amounts of lead [17]. Another study found that children exposed to >22.2 ppm of lead experienced an 8.6-point loss in reading scores on the word identification test compared to those exposed to only 5.99 ppm of lead [20]. Additionally, this study recognized a significant correlation between excessive lead exposure and deficits in grammatical reasoning. Children exposed to >22.2 ppm of lead on average answered 2–3 more questions incorrectly than those exposed to only 5.99 ppm of lead [20], further suggesting the possible consequences of lead exposure on language skills.

Lead exposure has also been correlated with language impairment in most adults, specifically, with difficulties in the expression of ideas. One study found that lead smelter workers experienced greater confusion when expressing an idea compared to non-exposed workers. In fact, workers exposed to >40 μg/dL obtained a score 10 points higher, on average, on confusion assessments compared to non-exposed workers, demonstrating lead may play role in altering language ability [24]. This also supports the hypothesis that lead exposure may play a part in furthering autistic comorbidities due to its ability to increase language difficulties.

### 3.4. Social Withdrawal

Social withdrawal, the act of avoiding others, is often correlated with excessive lead exposure [25,26,27]. A prior study, consisting of 194 pairs of children and their mothers, utilized the standard child behavior checklist to identify problematic behaviors subsequent to lead exposure. The prevalence of social withdrawal increased by 1.4% in high-lead-exposed children with a lead blood level >15 μg/dL (ages 2–3 years) compared to their low-lead-exposed counterparts with a lead blood level <15 μg/dL. This further supports an inverse relationship between performance on social evaluations and lead exposure [25]. Steering clear of friends and families is not the only sign of withdrawal; in fact, disinterest in normal activities is also common among lead-exposed children.

Another study investigated the relationship between lead exposure and the development of behavioral issues in infants using the Behavior Rating Scale (BRS) of the Bayley Scales of Infant Development. The BRS tests a variety of areas including distractible behaviors, disinterest in behaviors, social withdrawal, and propriety of movement. Children with elevated lead levels with a mean blood level of <25 μg/dL obtained a mean score 14.1 points lower than their lowly exposed counterparts in terms of the orientation–engagement factor assessing the extent of fear, withdrawal, as well as disinterested behaviors [27]. Antisocial behavior is commonly accompanied by anxiety of meeting others. Another study using the Behavior Rating Inventory of Executive Function (BRIEF) and the Conners’ attention deficit hyperactivity disorder (ADHD)/Diagnostic Statistical Manual for Mental Disorders found that anxiety, shyness, as well as general social problems were highest within children exposed to the greatest amount of lead (an average of 18.71 μg/dL) [26]. This further suggests lead may play a role in decreasing an individual’s social interaction capacity [25,26,27]. The prevalence of social withdrawal, loss in intelligence scores, decline in memory, and language difficulties in lead-exposed individuals further suggest the probable role of lead in furthering comorbidities associated with autism. While lead has been scientifically proven to cause these comorbidities, the presence of these comorbidities does not necessarily mean that they were induced by lead.

## 4. Mechanisms of Action

The accumulation of lead in the body has various consequences on the functioning of the central nervous system (CNS) [7,9,11,28,29,30,31,32,33,34,35]. Previous studies have highlighted alterations in several neurological mechanisms subsequent to lead exposure [7,9,11,28,29,30,31,32,33,34,35]. Prior research has emphasized the role of proper molecular structure in the adequate functioning of neurological mechanisms [36]. Indeed, several neuropsychiatric illnesses are associated with inconsistencies of molecular pathways, specifically, alterations within neurons and synapses [37]. Autistic individuals have been shown to possess a variety of dysfunctionalities within their dopaminergic, cholinergic, and glutamatergic systems [37]. Along with several other neurological dysfunctions, abnormal levels of dopamine transporters have been noted in autistic individuals [37]. In addition to the deterioration within the dopaminergic system, autistic individuals have been shown to possess restricted interests and decrements in energy metabolism associated with changes in the cholinergic system [38]. Additionally, reduction in glutaminergic neurotransmission has been demonstrated in autistic individuals [39].

Metals have been shown to induce these transformations and dysfunctionalities within neurological systems [30]. Lead, in particular has been associated with the alteration of the release and reuptake of several neurotransmitters [30]. Overall, changes among the cholinergic, dopaminergic, serotonergic, and glutamatergic systems are common among lead-exposed individuals [7,9,11,28,29,30,31,32,33,34,35]. Alterations within these neurological systems have been associated with several autistic symptoms, including deficits in behavior, decrements in learning and cognitive abilities, reading and writing impairments, memory loss, changes in the stress response, as well as increased anxiety [13,14,15].

### 4.1. Impact on Cholinergic System and Energy Metabolism

Amid many effects, lead has been shown to hinder the release of acetylcholine (ACh) which is responsible for many cognitive and memory processes involving the hippocampus and the prefrontal cortex through the activation of its cholinergic and muscarinic receptors [40]. Moreover, lead has the ability to mimic calcium in binding to proteins and interfere with calcium-requiring processes [30]. Lead has been shown to prevent calcium ions from entering the axon terminal, inhibiting the release of acetylcholinesterase (AChE). AChE regulates ACh levels, and the lack of its release induces acetylcholine accumulation in the synapses. Lack of AChE release may cause several issues, including impaired cognitive ability and memory due to insufficient stimulation of the cholinergic and muscarinic receptors [9].

Prior studies have also highlighted lead’s role in interfering with the function of protein kinase C (PKC) within the cholinergic system [9,32,33]. Generally located in neural tissues, PKC is a serine/threonine protein kinase required for many processes such as converting short-term memories to long-term memories, cognition, regulation and synthesis of neurotransmitters, signal transduction, synaptic plasticity, and regulation of the astrocytic gene coding for glial fibrillary acidic protein (GFAP) inside the blood–brain barrier (BBB) [9,41,42]. The BBB is responsible for preventing toxins from entering the brain, and lead has shown to induce changes within the BBB, making the brain vulnerable to disorders, among other issues. In fact, irregular GFAP expression has been shown in low-lead-exposed rats, which is significant as astrocytes make up the majority of the BBB [9,41]. Altered GFAP expression may cause changes in astrocyte shape, activity, and cooperation. Any alterations within the BBB may be correlated with cognitive deficits [27,41]

Likewise, the translocation of PKC activity from the cytosol to the membrane of cells has been noted subsequent to lead exposure in rats, disrupting the cerebral microvascular function. Inadequate cerebral microvascular function as well as impaired GFAP expression may be correlated with neurodegeneration, further relating to cognitive and memory decline [9,32,33,41]. Furthermore, studies have shown that spontaneous PKC activation in capillary cells disrupted energy metabolism following lead exposure [7,31,43]. Particularly, activation of protein kinase C by lead inhibits Na^+^/K^+^-ATPase, an enzyme accountable for more than two-thirds of the brain’s energy disbursement/expenditure [38]. One study highlighted lower Na^+^/K^+^-ATPase activity rin ats exposed to 0.05–0.2% of lead acetate compared to unexposed rats [35]. Another study found a 54% decrease in ATP levels in lead-exposed animals [31]. A continuous stock of energy is needed to carry out various metabolic processes, and small decrements can induce several complications within the central nervous system, including impairment of memory, cognitive ability, and language associated with autism.

### 4.2. Impact on the Dopaminergic System

Dopaminergic neurons are primarily located in the midbrain within the substantia nigra pars compacta (SNc) and the ventral tegmental area (VTA) [44]. The connectivity and structure of the dopaminergic (DAergic) neurons within these regions is primarily concerned with executive functioning, which includes decision-making, social ability, and analytical skills [30,44]. Several other behaviors such as locomotion, motivation, and recognition ability are also associated with dopamine neurotransmission [30]. Children with ASD have been shown to have deficits in the development of the dopaminergic system. Moreover, imaging studies have shown significant alterations in DA synthesis and dopamine transporter (DAT) expression levels in autistic individuals [37]. Therefore, abnormalities within the dopaminergic system may be involved in ASD.

Interestingly, protein kinase C has also been shown to play a role in the dopaminergic system along with the cholinergic system [30]. Abnormal levels of dopamine (DA) as well as malfunctions within the dopaminergic system may induce deficits in cognitive and communicative ability, which are common in autistic individuals [30]. Moreover, DA neurotransmission is prevented by dopamine transporter, as it is involved in the reuptake of DA back to the presynaptic neuron. Prior research has identified PKC as a positive regulator of DA and dopamine transport, suggesting a role for PKC in increasing the levels of DA extracellularly (in the presynaptic neuron). Lead has been shown to spontaneously activate PKC, in turn, increasing the levels of DA within presynaptic neurons [30]. This over-sufficiency has been linked to several neurological issues affecting cognitive ability, memory, and social interaction such as Parkinson’s disease [30]. Autistic individuals have been shown to have increased DAT levels, negatively impacting their executing functioning [9]. Prior research has found exposure to PKC activators such as heavy metals altered DAT function and activity [37,44,45]. Previous studies have also noted that DAT function and structure were specifically altered in PKC-1- or PKC-2-abundant *Caenorhabditis elegans* subsequent to lead exposure, while PKC-deficient *C. elegans* remained resistant to DAT alterations. This further suggests lead’s role in altering dopaminergic function via PKC. Modifications within the dopaminergic system due to the presence of lead may result in neurological deficits concerning language and reading capability, memory, and social ability [35].

Other dopaminergic parameters have also been shown to be impacted by lead. Prior studies have shown that the activity of tyrosine hydroxylase, the limiting enzyme in dopamine synthesis, was significantly impacted by excessive lead exposure [35]. Studies have correlated imbalances in dopamine to several neurological deficits as well as disorders, such as Alzheimer’s disease, further suggesting a role for lead in initiating autistic symptoms.

### 4.3. Impact on the Glutamatergic and GABAergic Systems

Glutamate is an excitatory neurotransmitter in the brain that transmits signals between nerve cells. Glutamate also has an imperative role in learning and memory. In fact, autistic individuals have been shown to have reduced levels of glutamate in the striatum [46]. Along with decrements in the striatum, impairments within chromosome regions that encode glutamate receptors have been found in individuals with autism [46]. Therefore, anomalies within the glutamatergic system may be consistent with the development of ASD. Additionally, gamma aminobutyric acid (GABA) is an inhibitory transmitter involved in behavior, cognition, as well as the stress response. In fact, autistic individuals have been shown to have decreased expression of various GABA receptors in the brain. One study highlighted that GABA A and GABA B receptors were highly reduced in cerebellar regions of autistic individuals [39].

Furthermore, chronic lead exposure is followed by alterations in the glutaminergic system, along with the dopaminergic and cholinergic systems. One study found severe decrements in glutamate release in rats exposed to 40 μg/dL of lead, which could be correlated with its calcium dependency [39,47]. As highlighted previously, lead has the ability to prevent calcium from entering the axon terminal, in turn decreasing calcium levels. Calcium is required to stimulate glutamate release from astrocytes; therefore, a reduction in calcium levels impairs this process [47]. Additionally, lead can inhibit calcium channels, further interfering with glutamatergic neurotransmission [39]. Previous studies have consistently observed a diminution in presynaptic calcium-dependent glutamate and GABA release subsequent to lead exposure within the hippocampus [9,35,48]. Due to glutamate’s integral role in learning, altered levels of glutamate may induce reading, language, memory, and cognitive deficits and may be correlated with lowered intelligence scores corresponding to autism. In addition, modified GABA levels may induce anxiety and social withdrawal associated with autism, because of GABA’s role in eliciting an appropriate stress response.

Another study highlighted a decline in vesicular proteins including synaptophysin (Syn) and synaptobrevin (Syb) associated with the glutamatergic and GABAergic systems following lead exposure during synaptogenesis. Vesicular release was dramatically lower in lead-exposed individuals [49]. Vesicles store glutamate and GABA neurotransmitters; therefore, deficits in vesicular proteins such as Syn and Syb may inhibit glutamate and GABA release. Studies have also shown reduced excitatory presynaptic currents (EPSCs) and inhibitory postsynaptic currents (IPSCs) after lead exposure [47,49]. EPSCs and IPSCs depend on glutamine and GABA neurotransmission, further supporting lead’s interference with glutamate and GABA release. Furthermore, changes within the glutamatergic and GABAergic systems may involve cognitive and social deficits which are associated with autism.

### 4.4. Influence on the NMDA Receptor

*N*-methyl-d-aspartate receptor (NMDAR), primarily functional in the hippocampus, plays an important role in memory and learning. NMDAR is one of three glutamatergic receptors essential to central synapses [50]. Previous studies have shown a correlation between NMDAR inhibition and loss of memory. Long-term potentiation (LTP), a process in which synapse connection becomes fortified, is primarily responsible for processing new memories [50]. Prior studies have highlighted the poor performance of rats on memory tasks due to LTP deficits [50,51]. LTP decline has been correlated with NMDAR inhibition [50,51]. Recent studies have highlighted the prevalence of mutations within NMDA genes along with several other abnormalities of NMDA function in autistic individuals [52]. Indeed, various ASD animal models have demonstrated diminished scores in spatial navigation and contextual response tasks, also due to inherent decrements in NMDAR [52,53]. Therefore, NMDAR abnormalities may significantly be associated with ASD.

Moreover, lead is a noncompetitive antagonist of NMDAR and has been implicated in learning decline in rats [54]. Specifically, modifications in the ontogeny and zinc-binding sites of subunits of NMDAR have been described subsequent to lead exposure in previous studies [50,55]. NMDAR consists of a compulsory NR1 subunit as well as other subunits related to NR2 and NR3 families. Splice variants of NR1 induce changes in the typical traits of NMDAR [50,55]. Lead has been shown to induce changes in the expression of splice variants of NR1, negatively impacting the function of NMDAR. One study found that expression of the NR1 subunit at the protein level was decreased by 24% in rats exposed to 750 ppm of lead acetate and by 58% in rats exposed to 1500 ppm of lead acetate [56]. Furthermore, NMDAR malfunction causes LTP decline and memory impairment, supporting lead’s role in inducing memory deficits.

Members of the NR2 family include NR2A, NR2B, NR2C, and NR2D. NR2A and NR2B are the most copious within the hippocampus. During development, an essential process must occur in which the majority of NR2B-containing NMDARs change to NR2A-containing NMDARs [55]. Lead has been shown to increase NR2B levels, thus disrupting the balance of NR2 subunits and possibly inhibiting the developmental switch from NR2B-containing NMDARs to NR2A-containing NMDARs [55]. Additionally, NR2A and NR2B have distinct zinc-binding sites. Studies have disagreed upon the mechanism of inhibition at zinc-binding sites by lead (whether that be competitive or noncompetitive), but there is agreement on the fact that lead has the ability to interact with zinc at the zinc-binding sites of the NMDAR subunits. Changes in NMDAR expression may induce difficulties in reading, language, and intelligence scores, as NMDAR is involved in learning and memory [50].

## 5. Conclusions

This review highlights the connection between lead exposure and the furthering of comorbidities associated with autism. A more comprehensive understanding of mechanisms associated with ASD is clearly warranted, as the disorder continues to be widespread and increase in frequency, with families continuing to face emotional, health, social, and financial burdens. This includes, but is not limited to, reduction of work hours, request of medical care, speech therapies, as well as special counseling. Moreover, the root cause of autism/ASD etiology still remains unclear, but metal exposure has been shown to contribute to the impact of ASD. The scientific findings discussed in this review corroborate the occurrence of several autistic symptoms subsequent to excessive lead exposure, including a decline in intelligence scores, social interaction, memory, and language.

More evidently, recent studies have also emphasized certain characteristics of lead that may play a significant role in the furthering of autistic comorbidities, such as lead’s ability to alter optimal cholinergic, dopaminergic, GABAergic, and glutamatergic function. One of lead’s most important properties is its ability to interfere with calcium-mediated processes, thereby negatively impacting the cholinergic and dopaminergic systems. In addition, lead’s ability to alter the functioning and optimal levels of several neurotransmitters within these systems may cause detrimental effects. In fact, lead-exposed individuals experience reductions in acetylcholine, glutamate, GABA, and NMDAR levels, which results in the decline of their reading and language abilities, stress response, as well as memory. Overall, the cited scientific studies support lead’s probable role in altering the cholinergic, dopaminergic, GABAergic, and glutamatergic systems, with ensuing functional abnormalities. The cited studies also highlight how these anomalies often result in academic and social difficulties associated with comorbidities of autism.

Further studies must be conducted in regard to lead exposure and its impact on each individual’s neurological system, due to the small number of existing clinical studies. Specialized studies, in particular, must be performed focusing on lead exposure and certain aspects of various neurological systems in order to increase our knowledge of lead’s impact on neurological mechanisms, such as the specific effect of lead on GABA receptors. Currently, critical cases of lead poisoning are treated primarily in two ways. Firstly, chelation therapy, which involves the intake of oral tablets which bind to lead in the body and promoting its excretion via the urinary system. Secondly, ethylenediaminetetraacetic acid (EDTA) chelation therapy employs an injection to rid the body of lead. Both options are recommended to individuals with a blood lead level >45 μg/dL [8]. Depending on the level of exposure, the amount of time required to remove lead from the body varies. The greater the exposure, the longer the time needed for lead to leave the body and for health improvement to occur. The amount of time necessary for health improvement may range from months to years, depending on each specific case as well as on the level of previous lead exposure [8]. Studies focusing on ways to reduce and combat lead exposure must be conducted in order to prevent further increase in societal ASD. In addition, new methods of removing lead from the body should be investigated, as lead exposure remains a growing health concern.

Furthermore, as autism continues to be a common psychiatric illness, families need to be reminded of the sources and potential dangers of lead to prevent further damage. In order to increase awareness, seminars and adequate healthcare must be increased on a global scale, especially in areas with a high lead exposure risk. Additionally, as autism is only one of several psychiatric illnesses, studies determining the connection between lead exposure and other neurological conditions must be conducted, including on the impact of lead exposure on Alzheimer’s disease and Parkinson’s disease.

## Data Availability

Not applicable.
